# A Hard Case to Swallow: Crucifix Esophageal Foreign Body in a Nonverbal Patient

**DOI:** 10.7759/cureus.9286

**Published:** 2020-07-19

**Authors:** Jessica A Barlow, Neil P Larson, Jesse Wray, Rachel E Bridwell

**Affiliations:** 1 Emergency Medicine, San Antonio Uniformed Services Health Education Consortium, San Antonio, USA; 2 Emergency Medicine, Brooke Army Medical Center, Fort Sam Houston, USA

**Keywords:** dyspnea, esophageal foreign body, dysphagia

## Abstract

Delayed presentation of esophageal foreign bodies places patients at high risk for esophageal perforation and infection. In nonverbal patients as well as children and adults with other concomitant illnesses, it is important to consider a broad differential diagnosis for presentations with upper respiratory complaints. The authors present a case of a nonverbal, elderly woman who presented after several days of mild, dry cough and was ultimately found to have a large esophageal foreign body that had been present for an unclear amount of time.

## Introduction

With an incidence of 13 per 100,000 people, the most common esophageal foreign body in the adult population is a food bolus [1]. However, the true incidence of nonfood esophageal foreign body ingestions in adults is unknown. The adult population that is most associated with foreign body ingestion includes individuals diagnosed with psychiatric conditions, neurological or intellectual disability, and those seeking secondary gain [2]. Esophageal foreign bodies are more common in male patients, and the most common chief complaint when these patients present to the emergency department (ED) is dysphagia [3]. However, esophageal foreign bodies may also masquerade as dyspnea and upper respiratory complaints in these high-risk populations. Given the severe potential morbidity and mortality of esophageal foreign bodies, the emergency physician must be able to quickly identify and appropriately treat this vulnerable patient population. The authors present a case of an elderly, neurologically handicapped female with ingestion of a large crucifix of unknown duration.

## Case presentation

A 70-year-old female with a past medical history of severe dementia, who was nonverbal at baseline, was brought to the ED by her family for two-day duration of dry cough. During the time of a global pandemic, the family was concerned that the patient may have early signs of COVID-19. Upon presentation, vital signs were within normal limits including oxygen saturation on room air. Physical examination demonstrated an elderly patient who was in no apparent distress and at her baseline nonverbal state. Her airway was intact with coarse breath sounds auscultated throughout bilateral lung fields, and partially masticated food was readily apparent in her mouth. However, she was not drooling and was tolerating her oral secretions without difficulty.

**Figure 1:**
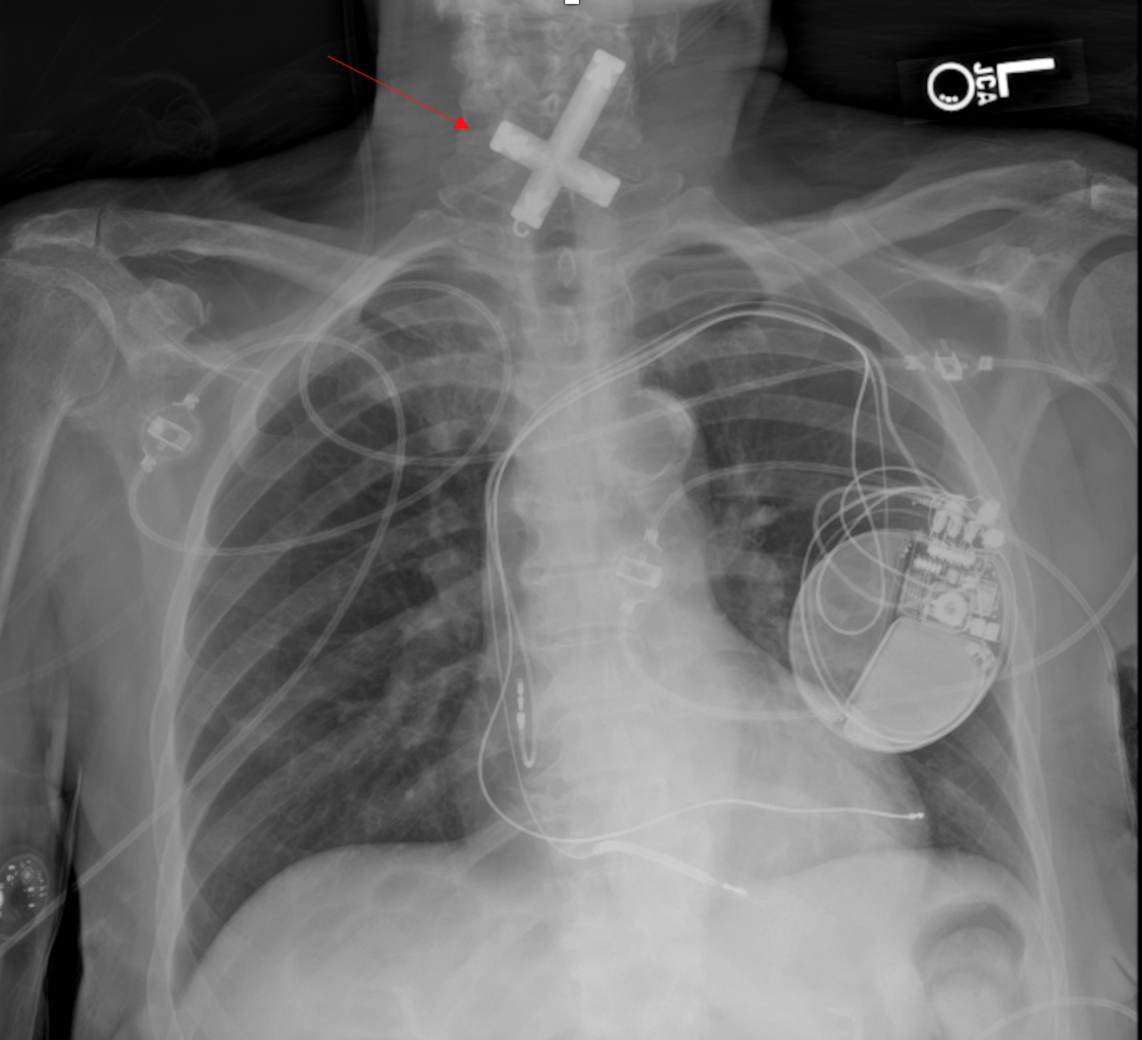
Posterioanterior chest radiography demonstrating a crucifix (red arrow) shaped object within the patient's neck.

**Figure 2:**
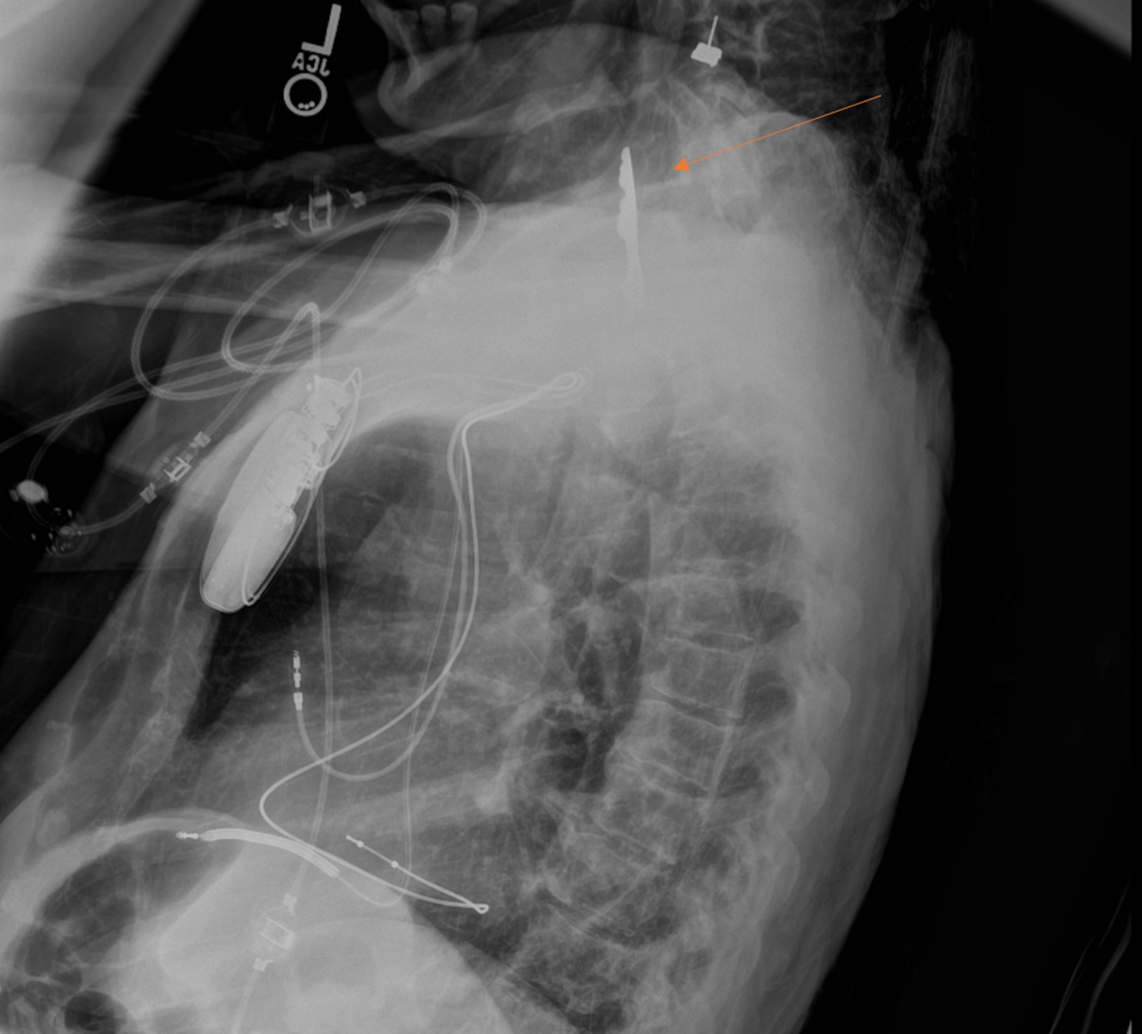
Lateral chest radiography demonstrating crucifix inferior to the epiglottis and posterior to the trachea consistent with a proximal esophageal foreign body (orange arrow).

A two-view chest radiograph was performed which revealed a crucifix-shaped foreign body measuring 8 cm by 5 cm in her esophagus (Figures 1, 2). Further discussion with the family revealed that the patient had persistently worn a crucifix pinned to her night gown in the past. While the securing pin was found in the laundry several days prior to presentation, it was unclear when the patient had ingested the object.

The patient was transferred to an outside hospital for expedited endoscopic extraction with gastroenterology where the foreign body was removed without complication. During hospitalization, the patient developed increasing leukocytosis and worsening consolidation on subsequent chest radiographs consistent with aspiration pneumonia. She was started on intravenous antibiotics as an inpatient and transitioned to oral antibiotics prior to discharge after persistent clinical improvement. After evaluation with speech pathology, the patient tolerated progressive advancement of her diet. The patient was ultimately discharged to a skilled nursing facility without any further complications.

## Discussion

Up to 80% of esophageal foreign bodies in adults may ultimately pass spontaneously [4]. However, esophageal foreign bodies remain a medical emergency warranting prompt evaluation as they are associated with high rates of morbidity and mortality. Multiple feared complications exist which vary based on the physical aspects of the object itself, the number of objects ingested, and the duration of ingestion. If not diagnosed and treated in a timely manner, patients are at risk of esophageal perforation, mediastinitis, vascular insult, and fistula formation among many others complications. The rates of complications increase significantly after 24 hours of foreign body presence [4]. Concomitant aspiration pneumonia in the setting of esophageal foreign body should be considered, with a higher index of suspicion in the intellectually disabled or nonverbal population. Patients who develop pneumonia following a recent esophageal foreign body and possible aspiration event in the proper clinical context should be treated with antibiotics providing appropriate pulmonary coverage, while recent recommendations suggest that oropharyngeal antibiotic coverage is not necessary [5,6]. Mortality related to esophageal foreign body complications is estimated to be as high as 20% [7]. Plain film radiography with posterioanterior and lateral chest imaging is the first step in workup; however, neck and abdominal films may also be of assistance [1]. Because radiography has several advantages including ease of access, ability to be rapidly obtained, low cost, and low radiation exposure, it is designated as the first choice imaging modality. However, not all foreign bodies are radiopaque and can be missed with radiography. If needed, computed tomography may better visualize and locate foreign bodies and assess for real-time complications.

Expedient removal is crucial for emergency management of esophageal foreign bodies. Up to 20% of adult patients with esophageal foreign bodies will ultimately necessitate endoscopic intervention; 2.7% of whom may require surgical intervention for foreign body extraction [3]. While not all endoscopic intervention may be considered emergent, emergency physicians should know that either the inability to handle secretions (indicating a possible complete esophageal blockage) or the presence of disc batteries or any sharp foreign bodies in the esophagus do necessitate emergent intervention [1]. In the ED setting, any symptomatic patient with confirmed or highly suspected esophageal foreign body unresolved by emergency physician intervention warrants consultation with gastroenterology, otorhinolaryngology or other experienced endoscopic interventionalist.

## Conclusions

Given high potential morbidity and mortality, adults with esophageal foreign bodies presenting to the ED warrant prompt triage, workup and intervention. Emergency physicians should quickly recognize the signs and symptoms of high-risk patients and consult the gastroenterologist or appropriate endoscopic interventionalist in a timely manner to improve patient outcomes.
